# CT-Centered Multimodality Imaging of Arterial Wall Fragility in Acute Aortic Syndromes: A Narrative Review of Imaging Markers and Clinical Implications

**DOI:** 10.3390/jcdd13060221

**Published:** 2026-05-22

**Authors:** Manuela Montatore, Ruggiero Tupputi, Federica Masino, Michela Montatore, Eluisa Muscogiuri, Giuseppe Guglielmi

**Affiliations:** 1 Department of Clinical and Experimental Medicine, Foggia University School of Medicine, Viale L. Pinto 1, 71122 Foggia, Italy; manuela.montatore@unifg.it (M.M.); federicamasino@gmail.com (F.M.); 2 Radiology Unit, “M. Dimiccoli” Hospital, Viale Ippocrate 15, 70051 Barletta, Italy; rutudott@gmail.com; 3 Department of Electrical Engineering and Electronics, Polytechnic University of Bari, 70125 Bari, Italy; m.montatore1@studenti.poliba.it; 4 Radiology Unit, “A. Perrino” Hospital, SS 7, 72100 Brindisi, Italy; eluisa.muscogiuri@asl.brindisi.it

**Keywords:** arterial wall fragility, acute aortic syndrome, CT angiography, aortic aneurysm, dissection, intramural hematoma, aortitis, imaging-derived markers, multimodality imaging

## Abstract

Arterial wall fragility represents a unifying pathophysiological substrate underlying a broad spectrum of aortic diseases, including aneurysms, dissections, intramural hematoma, penetrating atherosclerotic ulcers, and aortitis. Rather than distinct entities, these conditions increasingly appear as interconnected manifestations of impaired wall integrity and maladaptive vascular remodeling. This narrative review provides a structured overview of the imaging correlates of arterial wall fragility from a CT-centered, multimodality perspective. Computed Tomography Angiography (CTA) remains the first-line imaging modality in acute settings, enabling rapid and comprehensive assessment of vascular anatomy, luminal integrity, and the presence of life-threatening complications. Complementary modalities, including magnetic resonance imaging and ultrasound, contribute additional information on tissue characterization and hemodynamic evaluation in selected stable patients, follow-up settings, or specific clinical scenarios. Across imaging modalities, specific features—such as false lumen patency, intramural hematoma characteristics, ulcer-like projections, aneurysm morphology, and periaortic inflammatory changes—have been reported as markers of wall instability. These imaging-derived findings may provide clinically relevant information beyond traditional diameter-based assessment and support more refined risk stratification. Emerging approaches, including artificial intelligence, radiomics, computational modeling, and advanced MRI techniques, are expanding the role of imaging toward quantitative evaluation. However, their routine clinical implementation still requires standardization and prospective validation. Overall, a CT-centered multimodality imaging strategy may support a more comprehensive assessment of arterial wall fragility and contribute to individualized clinical decision-making in patients with aortic disease.

## 1. Introduction

Arterial wall fragility represents a unifying pathophysiological framework underlying a broad spectrum of aortic and arterial diseases, including aneurysms, dissections, intramural hematoma, penetrating atherosclerotic ulcers, and inflammatory aortopathies [1,2]. Although traditionally classified as distinct entities, increasing experimental and imaging evidence supports their interpretation as interconnected manifestations of impaired wall integrity and maladaptive vascular remodeling, often coexisting along a dynamic disease continuum [3,4,5].

The normal arterial wall is a highly organized and biologically active structure composed of endothelial cells, vascular smooth muscle cells, and extracellular matrix components—primarily elastin and collagen—which together ensure mechanical strength, elasticity, and adaptive response to pulsatile hemodynamic stress [6,7]. This balance is maintained through tightly regulated processes of matrix turnover, repair, and cellular signaling. Disruption of these mechanisms—due to aging, hypertension, atherosclerosis, genetic predisposition, or inflammatory activation—leads to progressive structural weakening, reduced biomechanical resilience, and increased susceptibility to acute wall failure [8,9,10]. From a pathophysiological standpoint, the clinical manifestations of arterial wall fragility depend on the predominant mechanism and spatial distribution of wall injury. These include progressive dilation (aneurysm formation), intimal disruption with medial delamination (arterial dissection), hemorrhage within the media (intramural hematoma), focal ulceration with penetration into the medial layer (penetrating atherosclerotic ulcer), and diffuse inflammatory damage (aortitis) [11,12,13]. Importantly, these entities frequently overlap or evolve from one to another, supporting the concept of a shared biological substrate rather than isolated disease processes [14,15].

Imaging plays a central role not only in diagnosis but also in the assessment of disease activity, risk stratification, and therapeutic decision-making. In the acute setting—particularly in suspected acute aortic syndromes—rapid and accurate imaging is critical for patient survival. Computed tomography angiography (CTA) has therefore become the first-line modality due to its wide availability, high spatial resolution, and ability to provide comprehensive evaluation of the aortic lumen, wall, branch vessels, and perivascular structures within a single acquisition [6,8]. Current guideline-based approaches emphasize the pivotal role of CTA in the emergency diagnostic pathway, particularly for detecting life-threatening complications such as rupture, malperfusion, and impending instability. However, a comprehensive assessment of arterial wall fragility increasingly requires a multimodality imaging approach. In selected unstable patients, echocardiography may provide sufficient information for urgent surgical decision-making when immediate CTA is unavailable or unsafe. While CTA remains the cornerstone in emergency settings, magnetic resonance imaging offers additional value in tissue characterization, functional assessment, and longitudinal follow-up, whereas ultrasound provides hemodynamic and screening information in selected vascular territories [10,12]. Current European Society of Cardiology (ESC) guidelines strongly emphasize the central role of multimodality imaging in the diagnostic and therapeutic management of acute aortic syndromes, particularly highlighting the need for rapid integration of anatomical and functional information in emergency settings. In this context, a multimodality approach is not merely complementary but essential for accurate diagnosis, risk stratification, and treatment planning. Recent evidence further reinforces this paradigm. The integration of computed tomography, magnetic resonance imaging, and echocardiography significantly improves diagnostic accuracy and clinical decision-making in acute aortic syndromes, particularly in complex or equivocal cases [13,14]. These findings support the concept that arterial wall fragility cannot be adequately characterized using a single imaging modality alone but rather requires a comprehensive and integrated diagnostic framework.

The integration of these modalities allows a more complete characterization of both structural and biological aspects of arterial wall disease. In this context, the present review aims to provide a structured and critical overview of imaging findings associated with arterial wall fragility, with a CT-centered but multimodality perspective. Particular emphasis is placed on imaging-derived features associated with wall instability, diagnostic pitfalls, and the clinical implications of imaging findings. Furthermore, emerging approaches, including advanced MRI techniques and artificial intelligence-based analysis, are discussed to highlight their potential role in improving risk stratification and moving beyond purely descriptive imaging. MRI does not represent a first-line modality in acute emergency settings but provides complementary value in selected and stable patients. Rather than providing a formal systematic synthesis or meta-analysis, this narrative review aims to offer a clinically oriented framework linking selected imaging findings with plausible pathophysiological mechanisms and their reported clinical implications. The goal is not to establish new quantitative risk estimates, but to summarize how CT-centered multimodality imaging may help recognize patterns of arterial wall instability across acute and chronic aortic disease.

### 1.1. Pathobiology of Aortic Wall Fragility

Arterial wall fragility reflects a multifactorial process characterized by extracellular matrix degradation, cellular dysfunction, and maladaptive vascular remodeling. The medial layer of the aorta, composed of concentric elastic lamellae interspersed with vascular smooth muscle cells, is essential for maintaining tensile strength and elastic recoil under pulsatile stress. Progressive elastin fragmentation, collagen disorganization, and smooth muscle cell apoptosis or phenotypic switching lead to impaired structural integrity and reduced capacity for repair [15,16].

Matrix metalloproteinases, particularly MMP-2 and MMP-9, play a key role in extracellular matrix degradation and are upregulated in response to inflammatory and hemodynamic stimuli. Their activity, combined with macrophage infiltration and chronic inflammation, promotes progressive weakening of the arterial wall [17]. Systemic hypertension further exacerbates these processes by increasing wall stress and favoring focal points of mechanical failure. In genetically predisposed individuals, including those with connective tissue disorders, intrinsic defects in matrix architecture accelerate these mechanisms and predispose to earlier and more aggressive diseases. The phenotypic expression of arterial wall fragility depends on the interplay between matrix degradation, inflammation, and compensatory remodeling [18,19]. This results in a spectrum of conditions ranging from aneurysmal dilation to acute dissection, intramural hematoma, penetrating ulcer, and inflammatory aortitis. These entities should therefore be interpreted as different manifestations of a shared pathobiological continuum rather than isolated diseases. From an imaging perspective, these underlying mechanisms translate into specific morphological patterns that can be detected and quantified. A CT-centered approach enables rapid identification of structural abnormalities associated with instability, while complementary imaging techniques may provide additional insights into tissue composition and disease activity. Accordingly, this review focuses on the integration of imaging findings with pathophysiological mechanisms, with the aim of improving diagnostic accuracy, risk stratification, and clinical management across the spectrum of arterial wall disease [20].

### 1.2. Clinical Translation of Arterial Wall Fragility

Beyond its pathophysiological relevance, arterial wall fragility has important implications for clinical decision-making. Several imaging features—such as rapid aneurysm growth, saccular or eccentric morphology, intramural hematoma thickness, ulcer-like projections, false lumen patency, and periaortic inflammatory changes—have been associated in the literature with disease progression, rupture risk, malperfusion, or need for urgent intervention.

However, these features should not be interpreted as isolated predictors. Rather, they should be integrated with clinical presentation, aortic location, patient age, comorbidities, genetic background, hemodynamic status, and guideline-based diameter or growth thresholds. Current clinical strategies are progressively moving from a purely diameter-based approach toward a broader risk assessment model incorporating anatomical, morphological, functional, and biological markers.

In this context, imaging-derived indicators of wall instability may help refine surveillance intervals, identify patients requiring closer follow-up, and support multidisciplinary decisions regarding surgical or endovascular intervention, particularly in borderline or complex cases. This review, therefore, considers arterial wall fragility as a dynamic imaging concept that may complement, but not replace, established clinical and guideline-based criteria.

## 2. Literature Search Strategy

This manuscript was designed as a narrative review. A targeted literature search was performed in PubMed, Scopus, and Embase to identify relevant peer-reviewed articles, reviews, and guideline documents published between January 2000 and January 2025. search terms included “acute aortic syndrome,” “aortic dissection,” “intramural hematoma,” “penetrating atherosclerotic ulcer,” “aortic aneurysm,” “aortitis,” “CT angiography,” “multimodality imaging,” “imaging markers,” “false lumen patency,” “aortic wall inflammation,” and “radiomics.”

Additional references were selected through manual screening of relevant articles and guideline documents. Studies were prioritized when they provided clinically relevant information on imaging findings, diagnostic performance, pathophysiological correlations, guideline-based management, or prognostic implications.

Because this was a narrative review, no formal systematic review protocol, PRISMA workflow, or meta-analytic synthesis was applied. The literature was summarized qualitatively, with emphasis on CT-derived imaging features of arterial wall instability and on the complementary role of MRI, echocardiography, ultrasound, PET-CT, and emerging quantitative techniques in selected clinical scenarios.

Representative anonymized imaging cases from the authors’ institutions were included for illustrative purposes only. They were selected to demonstrate typical imaging patterns of aneurysm, dissection, intramural hematoma, penetrating atherosclerotic ulcer, aortitis, and post-treatment findings. These cases were not intended to constitute an original clinical cohort or to provide outcome-based statistical evidence. Because this review is narrative in design, the search strategy was intended to support a representative and clinically relevant synthesis rather than exhaustive study identification or formal evidence grading.

## 3. Results

### 3.1. Arterial Dissection: CT Imaging of Wall Delamination

Arterial dissection arises from the separation of the arterial wall layers following an intimal tear, allowing blood to enter the media and form a false lumen that propagates longitudinally along the vessel [21]. CTA represents the gold standard in the acute setting, offering direct visualization of the intimal flap, precise differentiation between true and false lumens, and reliable assessment of longitudinal extent [22]. Beyond diagnosis, CTA provides critical information for clinical management, including hemodynamic consequences, branch-vessel involvement, and identification of complications. Key imaging features include:

- Differentiation of true and false lumens based on contrast enhancement and delayed opacification patterns;
- Assessment of false lumen thrombosis, either partial or complete;
- Detection of true lumen compression or collapse;
- Evaluation of dynamic or static malperfusion of branch vessels, which is essential for risk stratification.

Among these parameters, false lumen patency is an important CT-derived marker of persistent pressurization. Persistent flow within the false lumen has been associated with progressive aortic dilation, adverse remodeling, and increased need for reintervention [23,24,25]. CTA is also indispensable in identifying acute complications necessitating urgent intervention, including impending rupture, contained hematoma, rapid aortic expansion, or end-organ ischemia.

### 3.2. Aneurysm Formation: CT Evaluation Beyond Maximal Diameter

**Figure 1:**
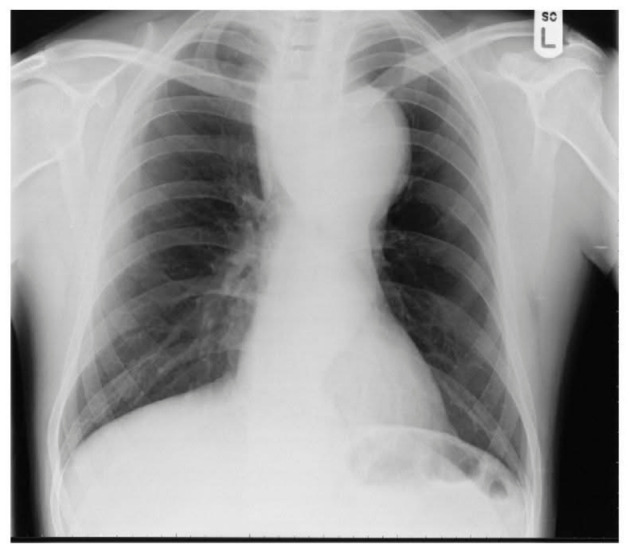
Anteroposterior chest radiograph demonstrating dilation of the aortic arch with contralateral tracheal displacement. Although chest X-ray cannot replace CTA for detailed assessment, it may provide an initial clue to underlying aortic pathology in patients presenting with acute chest pain, prompting further cross-sectional imaging.

**Figure 2:**
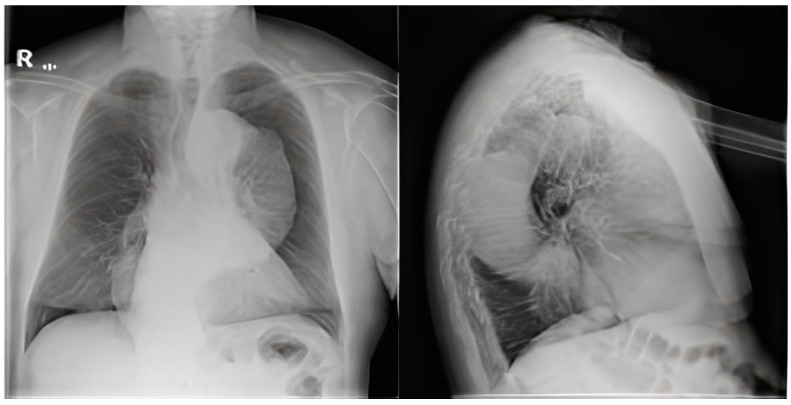
Chest radiograph of a patient presenting to the emergency department with dyspnea. The AP and LL projections reveal an incidentally detected thoracic aortic aneurysm, showing marked ectasia of the aortic arch with contralateral tracheal displacement.

**Figure 3:**
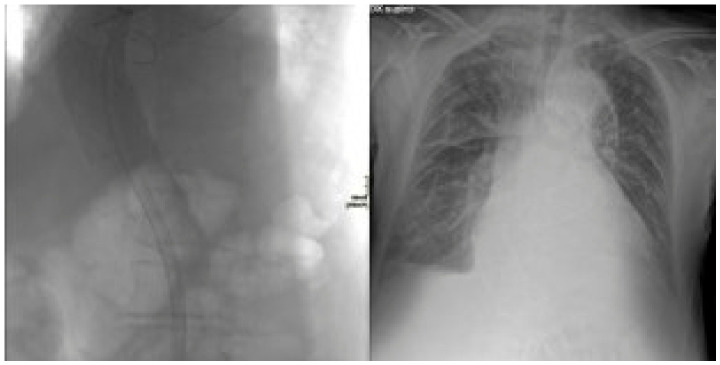
(**Left**): Digital subtraction angiography (DSA) of the thoracic aorta demonstrating endoleak within the aortic arch and descending aorta. (**Right**): Follow-up chest radiograph showing correct deployment and apposition of the endovascular stent. These findings highlight the complementary role of angiography and radiography in procedural guidance and post-treatment assessment.

**Figure 4:**
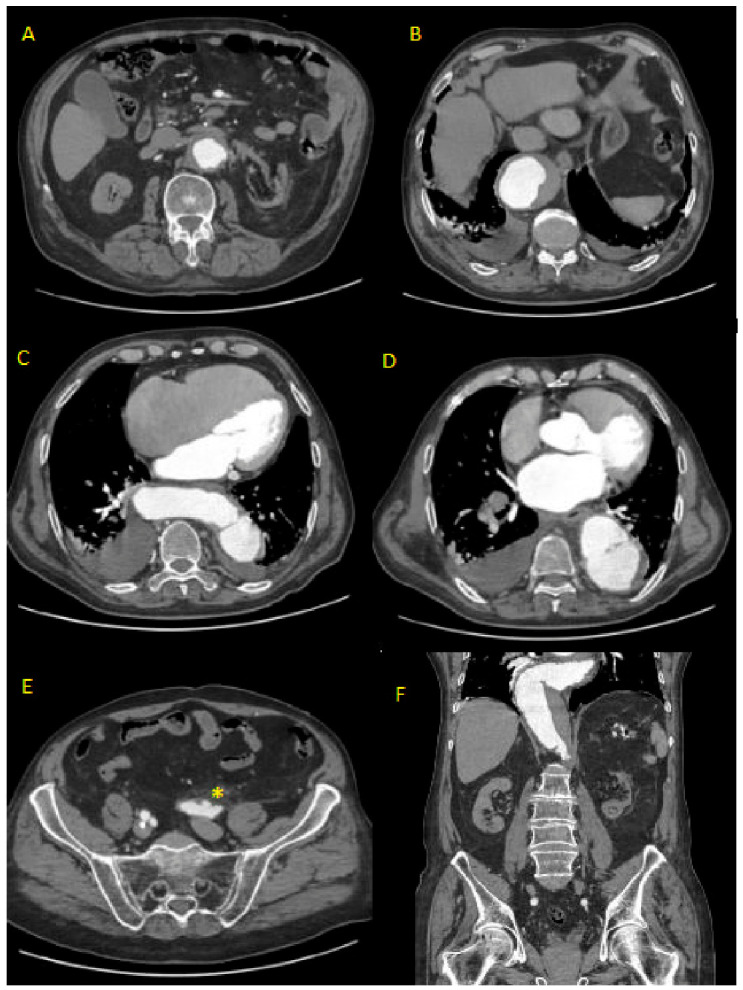
Contrast-enhanced CT angiography of the abdomen demonstrates complex aortic and visceral vascular pathology. Findings include a suprarenal aortic aneurysm (~48 mm) with atherosclerotic changes and focal ulcerations, associated infrarenal aortic dilation (~35 mm), and extension of calcified atherosclerosis to the iliac arteries. Additional features include occlusion of the inferior mesenteric artery with collateral revascularization, impaired renal artery opacification with bilateral renal atrophy, and focal dissection of the left common femoral artery. These findings illustrate the interplay between aneurysmal disease, atherosclerosis, and multilevel vascular involvement, highlighting the role of CTA in comprehensive assessment and treatment planning.

**Figure 5:**
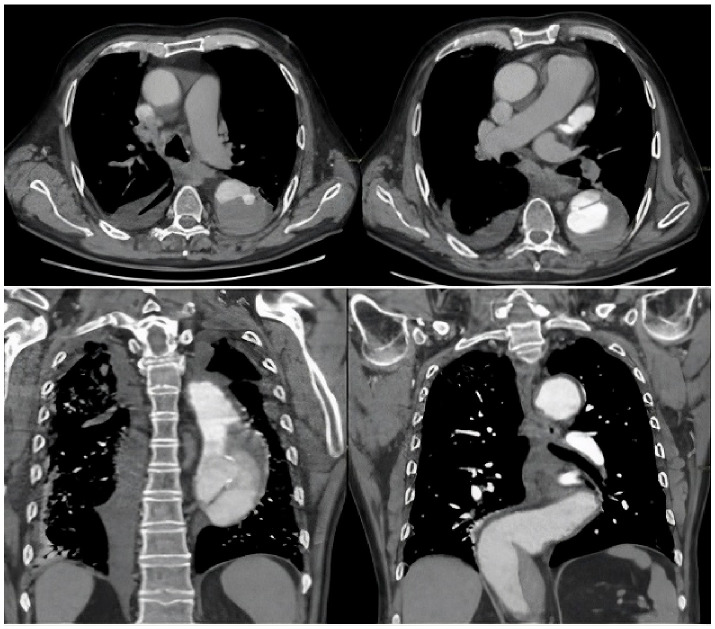
Contrast-enhanced CT angiography of the thoracic aorta demonstrating complex aneurysmal disease with S-shaped configuration, involving the aortic arch and descending aorta. Findings include ectasia of the ascending aorta (~40 mm), aneurysmal dilation of the arch (~55 mm), and severe dilation of the descending thoracic aorta (~70 mm) with extensive atherosclerotic plaque and luminal narrowing. A Stanford type B dissection is present distally, associated with intramural hematoma at the thoracoabdominal junction and partial celiac trunk thrombosis with distal re-opacification. Bilateral pleural effusions are also noted. These findings illustrate advanced multilevel aortic disease and its complications on CTA.

**Figure 6:**
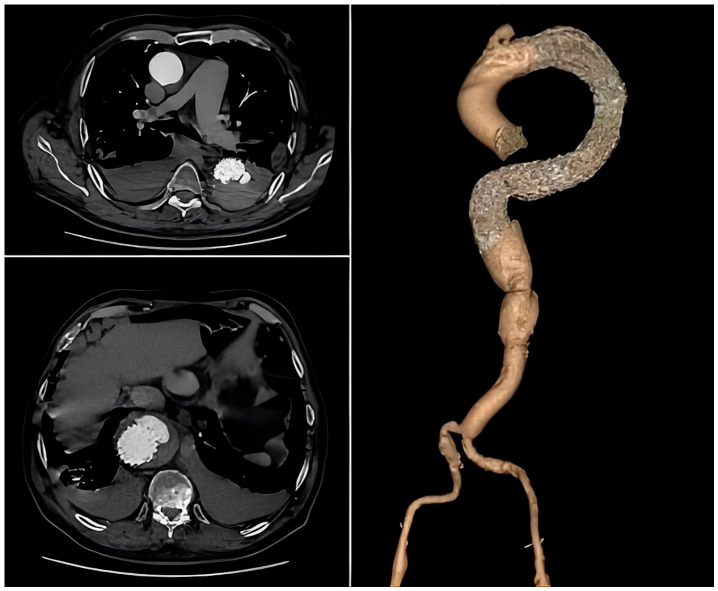
Follow-up CT angiography demonstrating post-procedural endoleak after endovascular aneurysm repair. Persistent contrast opacification outside the endograft is consistent with an endoleak, with residual aneurysmal sac perfusion. These findings highlight the role of CTA in post-treatment surveillance and detection of complications.

**Figure 7:**
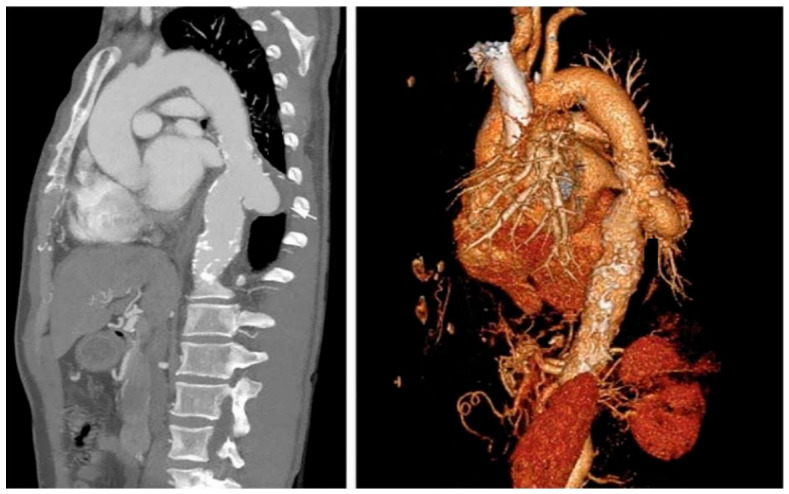
Follow-up CT angiography demonstrating post-procedural endoleak after endovascular aneurysm repair. Persistent contrast opacification outside the endograft is consistent with an endoleak, with residual aneurysmal sac perfusion.

An arterial aneurysm is classically defined as a permanent focal or diffuse dilation of a vessel exceeding 50% of its expected normal diameter [26]. In clinical practice, the abdominal aorta is considered aneurysmal at ≥3 cm, the ascending thoracic aorta at ≥4 cm, and the descending thoracic aorta at ≥3 cm [27,28]. Normal diameters vary by age, sex, and body surface area, ranging from 2.0 to 2.5 cm in the infrarenal abdominal aorta to 3.0–3.5 cm in the ascending thoracic aorta in adult males, with slightly lower values in females. CTA provides highly reproducible measurements of maximal diameter, length, volume, and spatial relationships to branch vessels, forming the cornerstone for surveillance protocols and surgical threshold planning [29]. While plain radiographs or ultrasound may occasionally reveal aneurysmal dilation incidentally, particularly in chest or abdominal projections, these modalities serve primarily as rapid, non-invasive screening tools in patients presenting with acute chest, back, or abdominal pain. They do not replace CTA for definitive characterization or treatment planning (Figure 1, Figure 2, Figure 3, Figure 4, Figure 5, Figure 6 and Figure 7) [30].

Representative CTA cases from the authors’ institutions are included to illustrate typical and high-risk imaging patterns of aneurysmal disease. These cases are intended for educational purposes and do not represent a prospective cohort or a statistical analysis of institutional outcomes. Current guidelines recommend intervention for asymptomatic abdominal aortic aneurysms generally at diameters of approximately 5.5 cm in men and 5.0 cm in women, although thresholds may vary according to patient-specific factors, anatomy, symptoms, and operative risk. For thoracic aortic aneurysms, intervention is generally considered at larger diameters, commonly around 5.5 cm for sporadic ascending aortic aneurysms, with lower thresholds in patients with heritable thoracic aortic disease, bicuspid aortic valve, rapid growth, family history of dissection, or planned concomitant cardiac surgery. Rapid interval growth should be reported according to disease-specific guideline thresholds, including ≥0.5 cm in 1 year or ≥0.3 cm/year over two consecutive years for thoracic aortic disease, with lower thresholds applied in selected high-risk conditions. Therefore, maximal diameter remains essential but should be interpreted together with morphology, growth rate, symptoms, and additional imaging features of instability [30]. However, maximal diameter alone is insufficient to fully predict aneurysm instability and rupture risk [31,32]. CTA provides a detailed evaluation of morphological and perivascular features reflecting localized wall fragility and biological activity, including:

- Rapid interval growth according to guideline-based thresholds;
- Eccentric or asymmetric dilation, concentrating mechanical stress on limited wall segments;
- Saccular morphology is associated with higher focal stress than fusiform aneurysms;
- Focal wall irregularities, blebs, or ulcer-like projections, indicative of microstructural compromise;
- Periaortic fat stranding or soft tissue changes, suggesting inflammation or acute remodeling.

These features reflect heterogeneous stress distribution, focal extracellular matrix degradation, and failure of adaptive remodeling mechanisms, all contributing to biological instability [33]. Intraluminal thrombus (ILT), readily assessed on CTA, has traditionally been considered protective. Contemporary evidence suggests that ILT may contribute to wall hypoxia, localized proteolytic activity, and inflammation, potentially influencing aneurysm growth and rupture risk [34]. Combining diameter-based thresholds with morphological and biological CT markers allows nuanced, patient-specific risk stratification, guiding surveillance intervals, timing of intervention, and decisions between elective versus urgent repair. In emergency or incidental scenarios, plain radiographs still provide rapid bedside information, accelerating CTA referral and early detection of potentially life-threatening pathology. Representative anonymized cases from the authors’ institutions are included for illustrative purposes only and were not analyzed as an outcome cohort. Future research should focus on prospective validation of imaging-derived markers and integration of artificial intelligence into clinical workflows to fully realize the potential of precision imaging in aortic disease.

### 3.3. Intramural Hematoma and Penetrating Atherosclerotic Ulcer

Intramural hematoma (IMH) is defined as hemorrhage confined to the medial layer of the arterial wall without an overt intimal tear detectable on initial imaging [35]. IMH is part of the acute aortic syndrome (AAS) spectrum, alongside classic dissection and penetrating atherosclerotic ulcers (PAUs), representing focal structural failure of the arterial wall. Pathophysiology involves rupture of the vasa vasorum or small intramural vessels, causing localized bleeding, wall stress, progressive weakening, and risk of secondary dissection or rupture [36]. On non-contrast CT, IMH appears as crescentic or circumferential hyperattenuating wall thickening, typically 5–15 mm in maximal axial thickness. Contrast-enhanced CTA reveals luminal narrowing, subtle wall irregularities, or progression into classic dissection, including development of secondary intimal flaps or pseudo-lumens. High-risk CTA features include:

- Maximal aortic diameter >50–55 mm;
- Periaortic hematoma;
- Ulcer-like projections;
- Persistent wall thickening.

Prognosis depends on location: ascending aorta (Stanford type A) involvement carries higher morbidity and mortality, while descending aorta (Stanford type B) may be managed conservatively with close imaging follow-up [37,38].

Penetrating atherosclerotic ulcers (PAUs) are focal intimal disruptions that extend into the media, usually in the context of advanced atherosclerosis [39]. CTA findings include:

- Contrast-filled outpouchings penetrating the aortic wall;
- Frequently associated with IMH, pseudoaneurysm formation, periaortic fat stranding, or adjacent inflammatory changes;
- Predominantly in the descending thoracic aorta.

**Figure 8:**
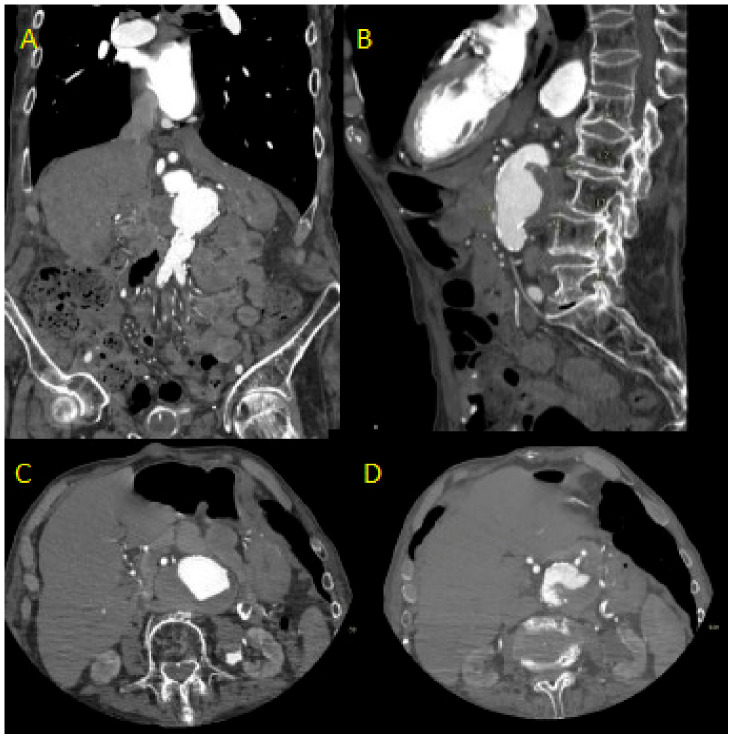
CT angiography of the abdominal aorta ((**A**): coronal; (**B**): sagittal; (**C**), axial and (**D**): axial at different level) demonstrating a complex juxtarenal aneurysm with aortobiiliac atherosclerosis. The aneurysm shows eccentric posterior mural thrombus with ulcerated components and circumferential thrombus (axial views), associated with a hyperdense crescent sign suggestive of intramural hemorrhage. The lesion extends anterior to the L3 vertebral body (sagittal view) with displacement of adjacent retroperitoneal structures. These findings illustrate morphological features associated with aneurysm instability on CTA.

**Figure 9:**
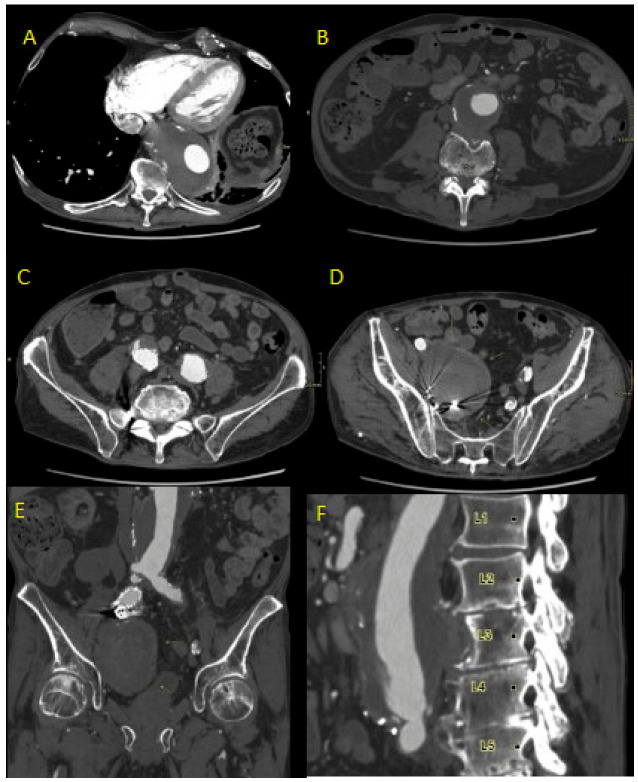
CT angiography after endovascular repair ((**A**): axial; (**B**–**D**): axial at different level; (**E**): coronal and (**F**): sagittal) demonstrating stent-graft deployment in the iliac axis with exclusion of the right internal iliac artery (L1–L5 level of the spine). Residual endoleak with persistent aneurysmal sac opacification is observed, along with diffuse thoraco-abdominal aneurysmal dilation and mural thrombus. These findings illustrate post-procedural remodeling and persistent perfusion as indicators requiring imaging surveillance.

**Figure 10:**
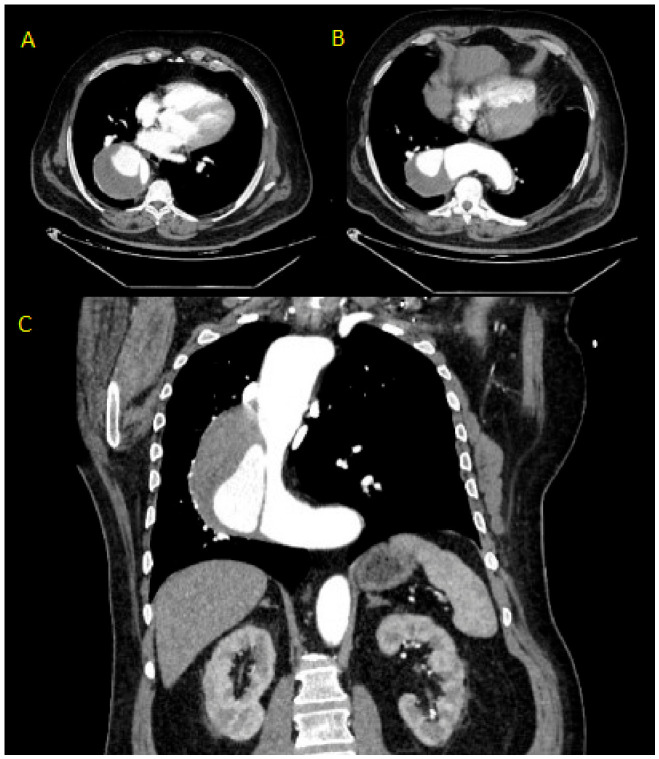
CT angiography ((**A**): axial; (**B**): axial at different leve; (**C**): coronal) demonstrating a saccular aneurysm of the descending thoracic aorta with eccentric mural thrombus and significant luminal compromise. Saccular configuration and thrombus heterogeneity are recognized imaging features associated with focal wall instability and increased rupture risk, supporting the role of CTA in risk stratification and management planning.

**Figure 11:**
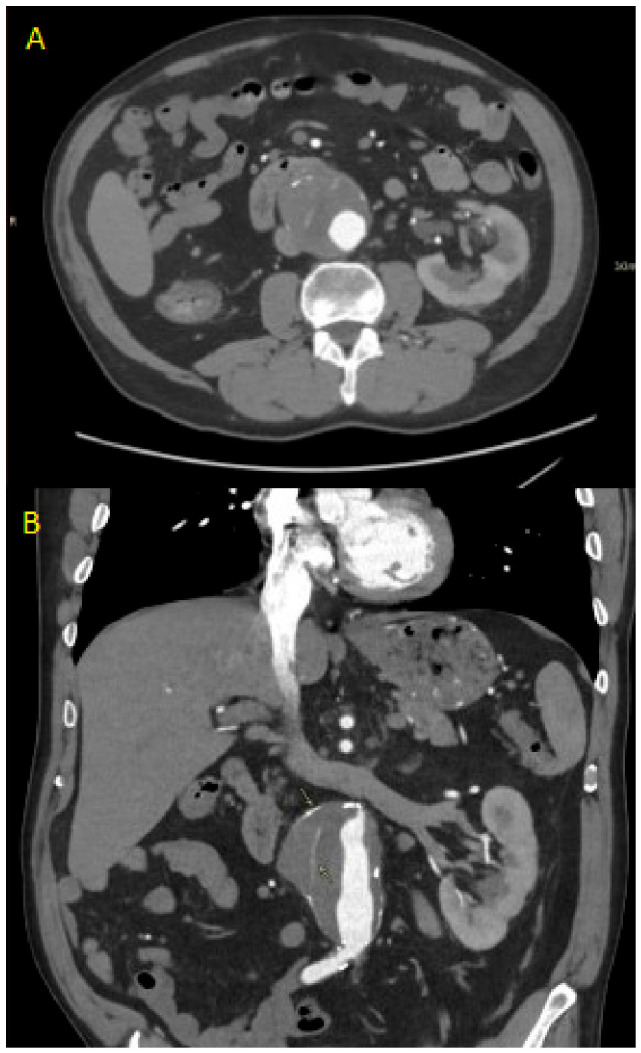
CT angiography ((**A**); axial; (**B**): coronal) showing a complex abdominal aortic aneurysm with interval growth, eccentric mural thrombus, and a residual intimal flap consistent with chronic dissection ((**B**), yellow arrows). These findings represent imaging markers of progression and wall instability, highlighting the role of CTA in acute evaluation and management planning.

**Figure 12:**
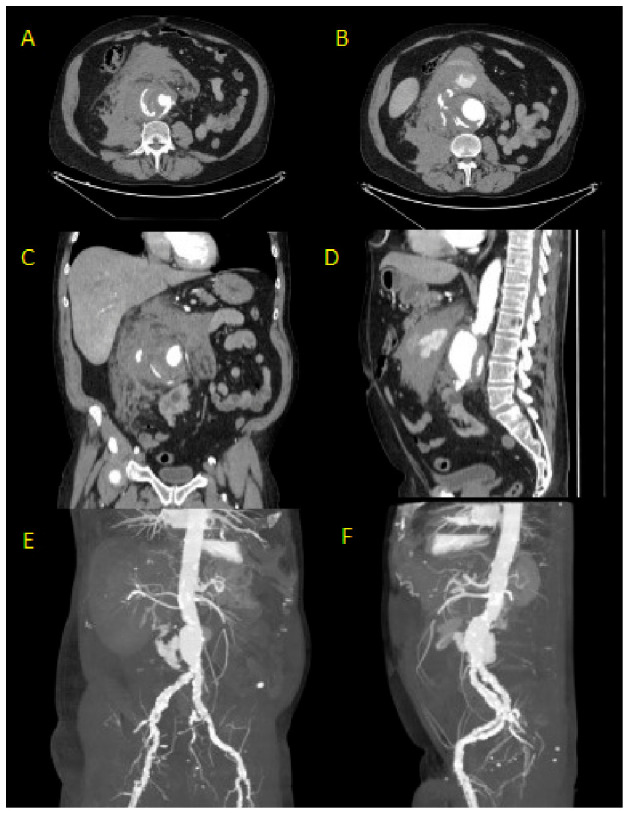
CT angiography ((**A**,**B**): axial at different level; (**C**): coronal; (**D**): sagittal and (**E**,**F**): vessel-window, VR image, in different position in rotation) showing a large dissected infrarenal abdominal aortic aneurysm with extensive mural thrombus and focal contrast extravasation. These features reflect advanced wall fragility and are highly suggestive of impending or active rupture, highlighting the role of CTA in emergency assessment and management.

**Figure 13:**
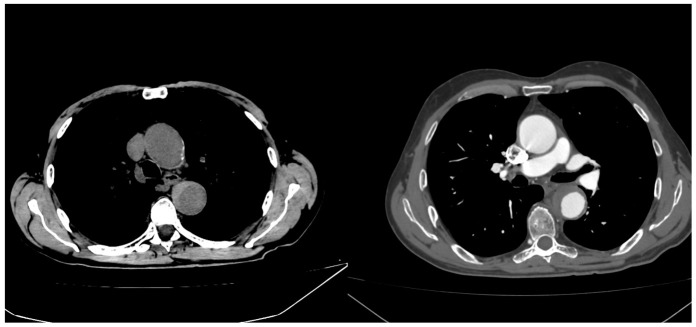
Emergency CT angiography ((**left**): non-contrast phase; (**right**): arterial phase) demonstrates a fusiform aneurysm of the descending thoracic aorta, with intraluminal hyperdensity on non-contrast images and active contrast extravasation on arterial-phase imaging. These findings are consistent with acute hemorrhage and aneurysmal instability, underscoring the pivotal role of CTA in detecting life-threatening complications.

**Figure 14:**
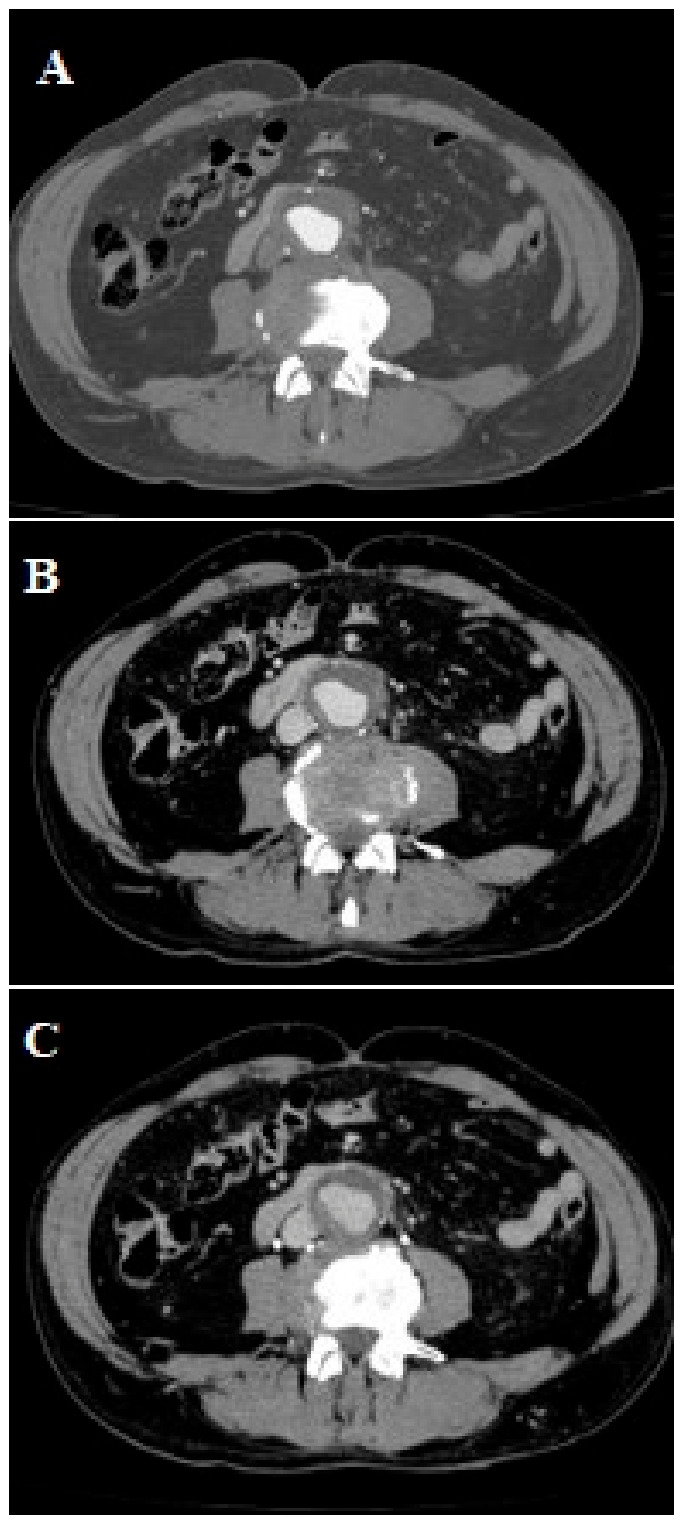
Multiphase CT angiography ((**A**–**C**): arterial-phase, axial plane at different level) demonstrates a fusiform infrarenal abdominal aortic aneurysm with irregular mural thrombus and focal wall bulging. These features reflect heterogeneous wall remodeling and potential instability, highlighting the value of multiphase CTA in aneurysm characterization and risk assessment.

**Figure 15:**
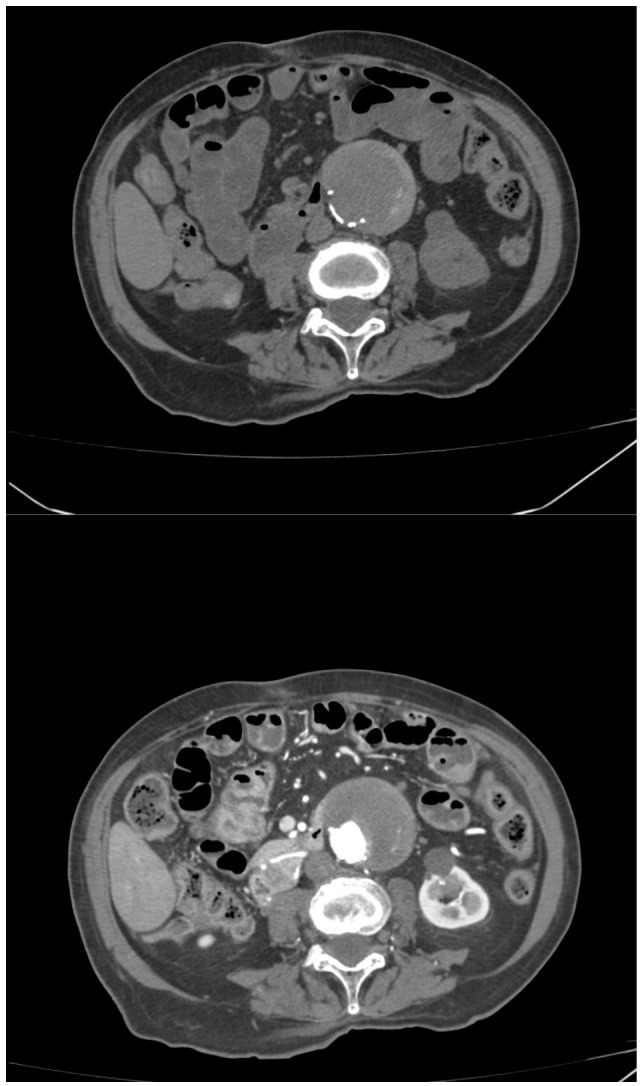
CT angiography showing a saccular infrarenal abdominal aortic aneurysm with eccentric mural thrombus and heterogeneous intraluminal components. The saccular morphology and thrombus characteristics represent imaging markers of focal wall instability and increased rupture risk.

**Figure 16:**
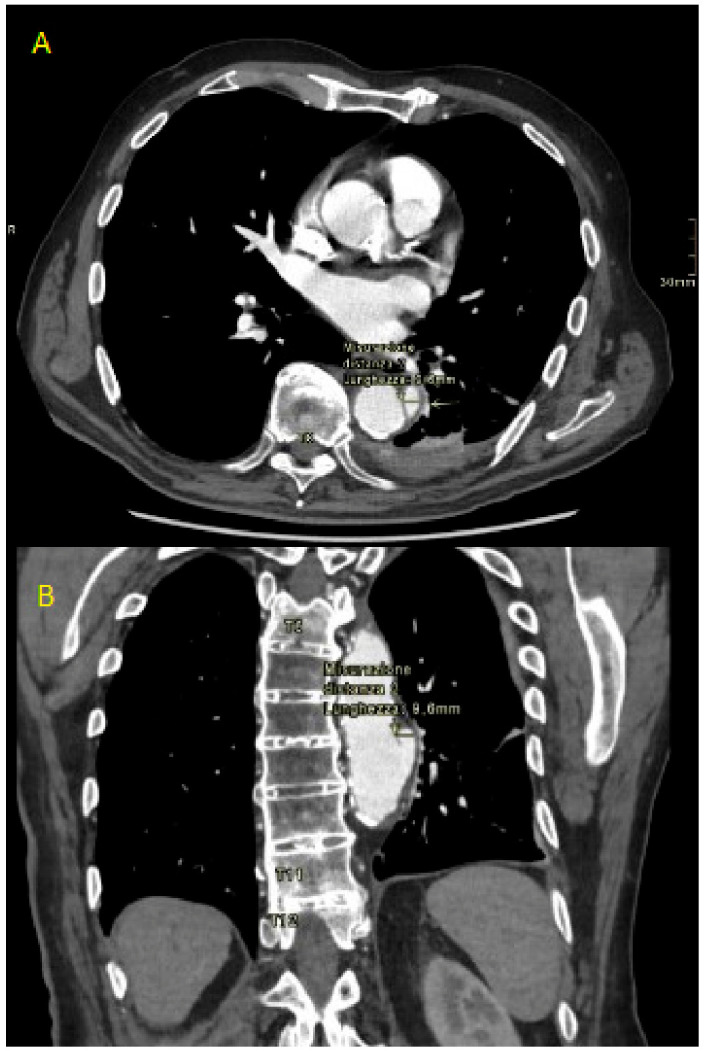
CT angiography ((**A**): axial; (**B**); coronal) demonstrating a penetrating aortic ulcer of the descending thoracic aorta associated with aneurysmal dilation and mural thrombus. The coexistence of ulceration and aneurysmal remodeling represents imaging markers of wall instability, highlighting the role of CTA in acute assessment.

PAUs can progress to dissection, rupture, or aneurysmal degeneration, especially with large lesion size (>20 mm depth), rapid expansion, or persistent mural hematoma. CTA is the first-line modality for diagnosis, risk stratification, and follow-up, guiding surgical or endovascular intervention for high-risk lesions, particularly in the ascending aorta or when progression is evident [40]. Follow-up strategies for IMH and PAU should be individualized according to aortic location, symptoms, maximum aortic diameter, ulcer depth and width, hematoma thickness, interval progression, and patient stability. Early repeat CTA is commonly performed in acute or equivocal cases to assess progression, development of ulcer-like projections, expansion of hematoma, or signs of impending rupture. Stable type B lesions may be managed conservatively with close clinical and imaging surveillance, whereas type A involvement, persistent pain, expansion, malperfusion, periaortic hematoma, or signs of rupture generally require urgent multidisciplinary evaluation. Thus, CTA provides both diagnostic and dynamic follow-up information, but imaging intervals should be adapted to clinical severity and guideline-based recommendations rather than applied as a fixed universal schedule (Figure 8, Figure 9, Figure 10, Figure 11, Figure 12, Figure 13, Figure 14, Figure 15 and Figure 16) (Table 1).

## 4. Discussion

Arterial wall fragility provides a useful conceptual framework for interpreting a range of aortic diseases, including aneurysm, dissection, intramural hematoma, penetrating atherosclerotic ulcer, and selected inflammatory aortopathies. Although these entities differ in presentation, natural history, and management, they may share overlapping mechanisms of wall injury, including medial degeneration, extracellular matrix remodeling, inflammation, altered hemodynamic stress, and impaired repair responses [41]. From an imaging perspective, this concept may help radiologists and clinicians interpret aortic disease not only as a luminal abnormality, but also as a manifestation of structural and biological wall vulnerability [42,43].

**Figure 17:**
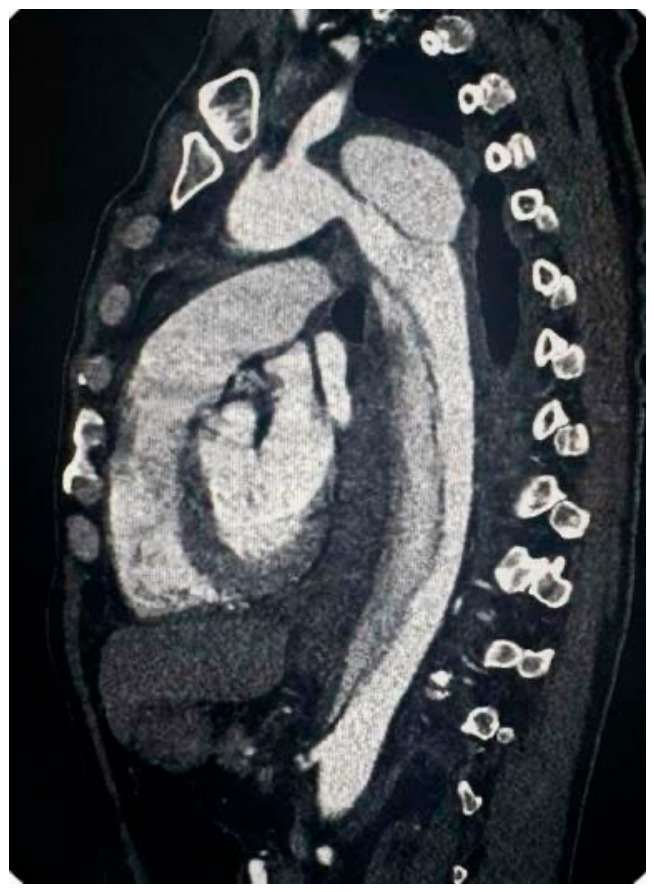
Sagittal chest CT of the descending thoracic aorta. The sagittal CTA image demonstrates a dissection of the descending aorta extending to the origin of the celiac trunk, with clear separation of the true and false lumens. In addition, a saccular aneurysm of the aortic arch is visualized, characterized by localized wall dilation and mural irregularities. These findings illustrate the complex interplay between dissection and aneurysmal formation, highlighting areas of increased wall stress and structural fragility. The case underscores the value of CTA in delineating both luminal and wall pathology, providing critical information for risk stratification and urgent therapeutic planning.

**Figure 18:**
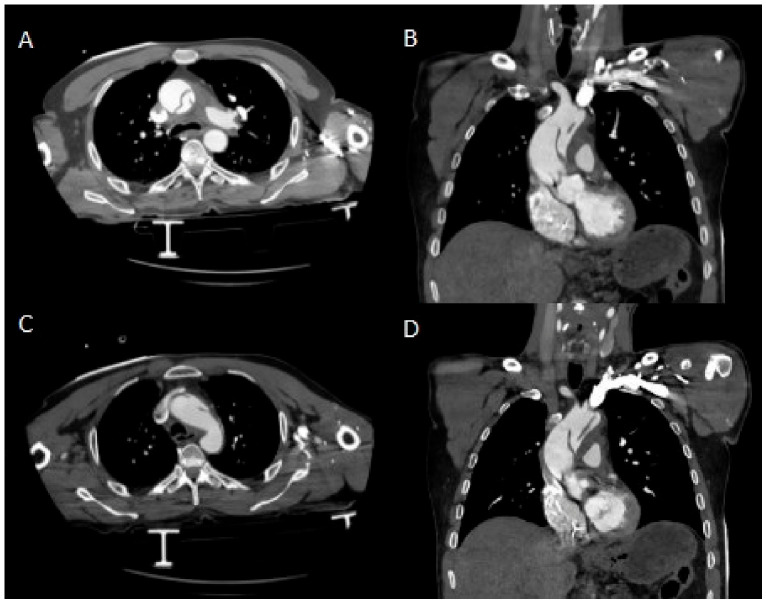
CT angiography of the thoracic aorta demonstrating acute aortic dissection. (**A**) Axial image at the aortic valvular plane shows the intimal flap originating at the level of the aortic root. (**B**) Coronal reconstruction confirms the extension of the dissection flap along the ascending aorta. (**C**) Axial image at the level of the aortic arch demonstrates the persistent intimal flap within the arch lumen. (**D**) Coronal reconstruction shows the dissection extending up to the origin of the left subclavian artery, with clear visualization of the true and false lumens.

**Figure 19:**
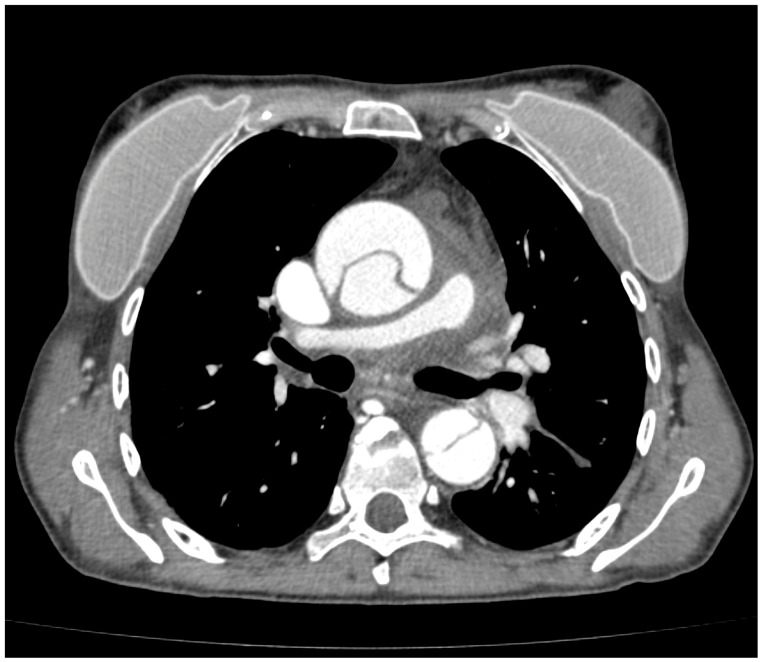
CT angiography demonstrating extensive Stanford type A aortic dissection extending from the ascending aorta to the iliac arteries, with branch vessel involvement and associated pericardial effusion. These findings reflect severe aortic wall instability with systemic and visceral complications.

**Figure 20:**
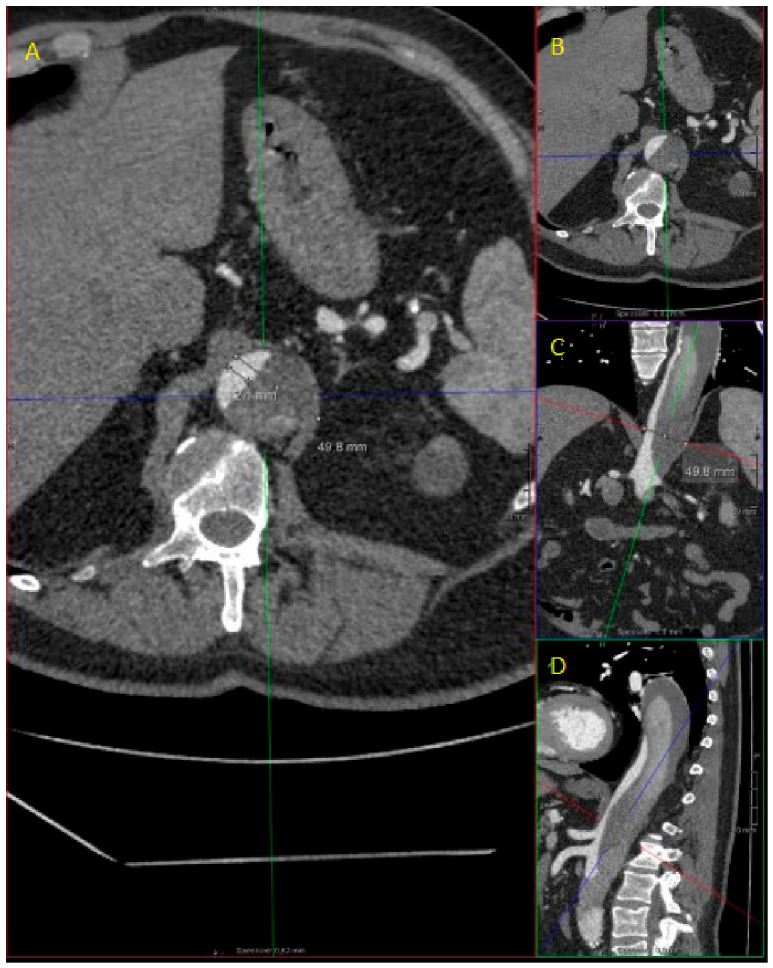
CT angiography ((**A**): axial; (**B**): axial at different level; (**C**): coronal and (**D**): sagittal) demonstrating chronic thoracoabdominal aortic dissection with persistent true and false lumen separation and associated mural hematoma. These findings reflect chronic wall remodeling and represent markers of ongoing wall fragility.

CTA remains the central imaging modality in most acute aortic scenarios because it is rapid, widely available, and able to evaluate the aortic lumen, wall, branch vessels, and periaortic tissues within a single examination. However, CTA findings should not be interpreted in isolation. Their clinical meaning depends on anatomical location, symptom severity, hemodynamic status, patient-specific risk factors, and guideline-based treatment thresholds. In this context, multimodality imaging may provide complementary information, particularly in stable patients, follow-up settings, and cases requiring tissue characterization or functional assessment (Figure 17, Figure 18, Figure 19 and Figure 20).

### 4.1. CTA in the Acute Evaluation of Aortic Disease

In suspected acute aortic syndrome, CTA plays a central role in confirming or excluding life-threatening aortic pathology. It allows direct visualization of dissection flaps, intramural hematoma, penetrating atherosclerotic ulcers, aneurysmal dilation, rupture, periaortic hematoma, and branch-vessel involvement. These findings are essential for early triage and for distinguishing uncomplicated presentations from cases requiring urgent multidisciplinary evaluation [44,45].

The value of CTA extends beyond diagnosis. By providing high-resolution multiplanar and three-dimensional assessment, CTA supports evaluation of disease extent, true and false lumen morphology, malperfusion, pericardial or pleural complications, and signs of impending rupture. These features may influence medical stabilization, surgical planning, endovascular strategy, and follow-up intensity. Nevertheless, CTA should be considered a decision-support tool rather than a stand-alone determinant of management, since treatment decisions require integration with clinical, surgical, and hemodynamic information.

Several CT findings may suggest increased wall instability, including false lumen patency, true lumen compression, ulcer-like projections, focal wall irregularity, periaortic hematoma, inflammatory soft-tissue changes, and rapid interval growth. The prognostic value of these features varies across disease entities and is not always supported by the same level of evidence. Therefore, structured reporting should describe these findings clearly while avoiding overinterpretation when longitudinal or outcome data are limited.

### 4.2. Classification Systems and Their Imaging Context

The Stanford and DeBakey classifications remain fundamental for the clinical management of aortic dissection. CTA allows rapid classification of dissections according to ascending aortic involvement and longitudinal extent, thereby supporting urgent differentiation between type A disease, which generally requires surgical evaluation, and type B disease, which may be managed medically, endovascularly, or surgically depending on complication status.

However, contemporary CT interpretation should not stop at classification alone. Within the same anatomical category, patients may show markedly different imaging patterns and clinical risks. In type B dissection, for example, false lumen patency, partial thrombosis, large entry tears, early aortic enlargement, true lumen compression, branch-vessel compromise, and periaortic hematoma may indicate a higher-risk phenotype. Similarly, in type A dissection, pericardial effusion, coronary involvement, aortic regurgitation, arch-vessel involvement, and malperfusion influence urgency and operative planning.

Thus, Stanford and DeBakey classifications remain clinically indispensable, but they are best interpreted together with CT-derived markers of disease extent, perfusion, wall morphology, and complications. This integrated approach may improve communication between radiologists, cardiologists, vascular surgeons, and cardiac surgeons, particularly in emergency settings.

### 4.3. CTA Beyond Diameter: Imaging Markers of Instability

Diameter remains a cornerstone of aortic risk assessment and intervention planning, particularly in aneurysmal disease. However, diameter alone may not fully capture focal wall weakness, biological activity, or short-term instability. For this reason, increasing attention has been directed toward additional imaging features that may refine risk assessment.

CTA-derived markers such as rapid interval growth, saccular or eccentric morphology, focal wall irregularities, blebs, ulcer-like projections, intraluminal thrombus characteristics, periaortic stranding, and mural enhancement may provide useful contextual information. In dissection, false lumen patency and true lumen compression may reflect persistent pressurization and altered perfusion. In intramural hematoma and penetrating atherosclerotic ulcer, hematoma thickness, ulcer dimensions, ulcer-like projections, and periaortic hematoma may suggest increased risk of progression or complication.

These findings should be interpreted as complementary markers rather than independent predictors. Their clinical value depends on disease type, aortic segment, symptoms, growth pattern, comorbidities, and therapeutic feasibility. In some settings, such as false lumen patency after type B dissection or rapid aneurysm growth, the association with adverse remodeling is relatively well established. In other settings, such as subtle wall irregularity or inflammatory periaortic changes, evidence remains more heterogeneous. Therefore, these markers may be most useful when incorporated into structured reports and multidisciplinary discussions rather than used as isolated decision thresholds.

### 4.4. Intramural Hematoma and Penetrating Atherosclerotic Ulcer as Models of Wall Instability

Intramural hematoma and penetrating atherosclerotic ulcer illustrate how aortic wall disease may progress even in the absence of a classic dissection flap. IMH reflects hemorrhage within the aortic media, whereas PAU represents ulceration of an atherosclerotic plaque extending into the medial layer. Both entities are part of the acute aortic syndrome spectrum and require careful imaging assessment because their clinical course may range from stabilization to rapid progression.

CTA is particularly useful for identifying IMH thickness and extension, ulcer-like projections, focal contrast outpouchings, periaortic hematoma, pleural effusion, and interval change. These features may help identify patients who require close surveillance or urgent intervention. However, the management of IMH and PAU is highly dependent on location, symptoms, aortic diameter, ulcer morphology, hematoma progression, and patient stability.

A key practical point is that IMH and PAU should not be described only as static findings. Serial imaging may reveal expansion, regression, development of ulcer-like projections, progression to classic dissection, pseudoaneurysm formation, or rupture. Therefore, CTA contributes both to initial diagnosis and to dynamic follow-up, while MRI may be useful in selected stable patients for tissue characterization and longitudinal assessment without repeated radiation exposure.

### 4.5. Inflammatory Aortopathies and Aortitis

Although aortitis is not classically grouped with acute aortic syndromes in all classifications, inflammatory aortic disease is relevant to the broader concept of arterial wall fragility. Inflammation may weaken the aortic wall through edema, inflammatory infiltration, destruction of elastic fibers, smooth muscle cell injury, and remodeling of the extracellular matrix. Over time, these mechanisms may predispose to aneurysmal dilation, dissection, stenosis, or rupture, depending on the underlying etiology and disease activity.

CTA can demonstrate structural manifestations of aortitis, including concentric or eccentric wall thickening, mural enhancement, periaortic fat stranding, soft-tissue infiltration, aneurysmal change, and complications such as contained rupture or pseudoaneurysm. These findings are particularly relevant in symptomatic or acute presentations, where CTA may rapidly assess both inflammatory wall changes and structural complications.

MRI and PET-CT may provide complementary information in selected patients. MRI may help characterize wall edema and mural inflammation, while PET-CT may support assessment of metabolic activity, especially in suspected large-vessel vasculitis or infectious aortitis. However, these modalities should be chosen according to clinical urgency, availability, renal function, radiation considerations, and the specific diagnostic question. In many acute settings, CTA remains the most practical first-line examination, while MRI and PET-CT may be more useful for problem solving, disease monitoring, and treatment response assessment.

### 4.6. Toward a CT-Centered Multimodality Framework

A CT-centered multimodality framework may be useful because different imaging modalities answer different clinical questions. CTA provides rapid anatomical definition and complication assessment. Echocardiography can provide immediate bedside information on proximal aortic involvement, aortic valve function, pericardial effusion, tamponade, and cardiac consequences, particularly in unstable patients. Ultrasound is valuable for abdominal aortic aneurysm screening and surveillance. MRI offers tissue characterization, flow assessment, and radiation-free follow-up in selected stable patients. PET-CT may contribute to the evaluation of inflammatory or infectious aortic disease.

The practical goal is not to use all modalities in every patient, but to select the appropriate modality according to the clinical scenario. In an unstable patient with suspected acute type A dissection, echocardiography may provide immediate information while CTA defines anatomy when feasible. In stable patients with chronic aortic disease, MRI may reduce cumulative radiation exposure and provide functional assessment. In suspected inflammatory aortopathy, PET-CT or MRI may complement CTA by assessing disease activity.

This approach may also improve reporting. A structured CTA report for suspected acute aortic syndrome should ideally include aortic segment involvement, maximal diameters, dissection entry site when visible, true and false lumen status, branch-vessel origin and perfusion, malperfusion signs, IMH thickness, PAU dimensions, periaortic hematoma, pleural or pericardial effusion, and signs of rupture or impending rupture. Such reporting may improve multidisciplinary communication and support consistent follow-up.

### 4.7. Emerging Quantitative Imaging: AI, Radiomics, 4D-Flow MRI, and Computational Modeling

Emerging quantitative techniques may further refine the assessment of arterial wall fragility, although most remain investigational or incompletely standardized. Artificial intelligence and deep learning methods have shown potential for automated aortic segmentation, diameter measurement, lumen classification, thrombus quantification, and volumetric assessment. These tools may reduce reporting time and interobserver variability, particularly in complex or longitudinal cases [46,47].

Radiomics may provide additional information by extracting quantitative texture and shape features from CT or MRI datasets. These features may reflect heterogeneity in thrombus, wall structure, inflammation, or remodeling that is not fully captured by visual interpretation. However, radiomics studies often differ in acquisition protocols, segmentation methods, feature extraction pipelines, and validation strategies. Therefore, their clinical translation requires external validation, reproducibility testing, and demonstration of incremental value over conventional imaging and clinical predictors.

Advanced MRI, including 4D-flow MRI, may provide insight into flow patterns, wall shear stress, vortex formation, and regional hemodynamic abnormalities. These parameters may be relevant in aneurysm progression, bicuspid aortic valve-associated aortopathy, chronic dissection, and post-repair surveillance. Computational modeling, including finite element analysis and wall stress mapping, may further support patient-specific biomechanical assessment. Nevertheless, these approaches should currently be viewed as promising adjuncts rather than established routine tools.

Future research should focus on prospective multicenter validation, standardized acquisition protocols, transparent algorithms, and clinically meaningful endpoints. The most valuable models will likely be those integrating imaging features with clinical variables, genetics, laboratory markers, and longitudinal outcomes. Until such validation is available, quantitative imaging should complement rather than replace established guideline-based decision-making.

### 4.8. Limitations of the Current Evidence and of This Review

Several limitations should be acknowledged. First, this is a narrative review and does not provide a formal systematic review, meta-analysis, or pooled estimate of diagnostic or prognostic accuracy. The literature was selected to support a clinically oriented synthesis rather than exhaustive evidence grading. Second, the evidence supporting different imaging markers is heterogeneous. Some markers are well established in specific contexts, whereas others are based on smaller studies, expert consensus, retrospective cohorts, or indirect pathophysiological reasoning.

Third, imaging protocols, terminology, measurement techniques, and follow-up intervals vary across institutions and studies, limiting direct comparison. Fourth, many advanced imaging approaches, including radiomics, artificial intelligence, 4D-flow MRI, and computational modeling, remain insufficiently standardized for routine clinical use. Finally, representative imaging cases included in this review are intended for educational illustration and should not be interpreted as an original outcome cohort.

Despite these limitations, a CT-centered multimodality approach may help organize the imaging evaluation of arterial wall fragility in a clinically meaningful way. By linking anatomical findings, wall-related features, complications, and complementary modalities, imaging can contribute to a more precise description, better communication, and individualized management planning in patients with acute and chronic aortic disease.

## 5. Conclusions

Arterial wall fragility can be interpreted as a unifying framework encompassing several acute and chronic aortic diseases, including aneurysm, dissection, intramural hematoma, penetrating atherosclerotic ulcer, and aortitis. In this setting, CTA remains the cornerstone imaging modality for acute evaluation because it provides rapid, high-resolution assessment of aortic anatomy, luminal integrity, branch-vessel involvement, and life-threatening complications.

Imaging-derived features such as false lumen patency, rapid interval growth, saccular or eccentric morphology, wall irregularities, intramural hematoma characteristics, ulcer-like projections, and periaortic inflammatory changes may help refine assessment beyond diameter alone. These findings should be interpreted as complementary markers of instability rather than independent predictors, and they should always be integrated with clinical presentation and guideline-based criteria.

MRI, echocardiography, ultrasound, and PET-CT provide additional value in selected clinical contexts, particularly for follow-up, tissue characterization, hemodynamic assessment, and inflammatory disease evaluation. Emerging tools such as artificial intelligence, radiomics, advanced MRI, and computational modeling are promising, but their routine use requires further validation. Overall, a CT-centered multimodality approach may improve the characterization of arterial wall fragility and support individualized decision-making in patients with aortic disease.

## Figures and Tables

**Table 1:** CT-derived imaging markers of arterial wall instability and their reported clinical relevance. This table is intended as a qualitative synthesis and not as a pooled quantitative estimate of prognostic effect.

Condition	CT Imaging Marker	Pathophysiological Meaning	Reported Association	Clinical Relevance
Aortic dissection	Persistent false lumen patency	Ongoing false lumen pressurization and impaired remodeling	Associated with aortic enlargement, adverse remodeling, and need for closer follow-up or reintervention	Supports surveillance planning and risk stratification
Aortic dissection	True lumen compression or collapse	Dynamic or static obstruction of branch-vessel perfusion	Associated with malperfusion syndromes and complicated dissection	May prompt urgent endovascular or surgical evaluation
Aneurysm	Rapid interval growth	Accelerated wall remodeling and structural weakening	Associated with increased rupture risk and guideline-based indication for repair	Influences the timing of intervention
Aneurysm	Saccular or eccentric morphology	Localized wall stress concentration and focal structural weakness	Considered higher risk than fusiform morphology in several clinical settings	May lower threshold for intervention
Aneurysm	Wall irregularity, blebs, or ulcer-like projections	Focal disruption of the wall surface or plaque penetration	Associated with local instability and potential progression	Supports closer follow-up or treatment planning
Aneurysm	Intraluminal thrombus burden or heterogeneity	Wall hypoxia, inflammation, and proteolytic activity beneath the thrombus	Associated with aneurysm growth and biological wall activity	Complements the diameter-based assessment
Intramural hematoma	Hematoma thickness and longitudinal extension	Medial hemorrhage and increased wall stress	Greater thickness and extension have been linked to progression, ulcer-like projection, or rupture.	Helps guide early reassessment and treatment strategy
Penetrating atherosclerotic ulcer	Ulcer depth, width, and associated IMH	Atherosclerotic plaque penetration into the media	Larger or progressive ulcers are associated with complications, including pseudoaneurysm, rupture, or dissection	Supports risk stratification and follow-up intensity
Aortitis	Wall thickening and mural enhancement	Active inflammatory infiltration and edema	May reflect disease activity, especially when progressive or associated with symptoms	Guides additional MRI/PET-CT assessment and treatment monitoring
Aortitis	Periaortic fat stranding or soft tissue infiltration	Perivascular inflammatory extension	Associated with active inflammation or acute complications in selected cases	Helps distinguish active from chronic disease patterns

## Data Availability

No new data were created or analyzed in this study. The clinical images included are fully anonymized and were obtained from routine clinical practice; they are not publicly available due to privacy and ethical restrictions.

## Abbreviations

The following abbreviations are used in this manuscript:

Abbreviation	Meaning
AAA	Abdominal Aortic Aneurysm
AI	Artificial Intelligence
CTA	Computed Tomography Angiography
DECT	Dual-Energy CT
IMH	Intramural Hematoma
MMP	Matrix Metalloproteinase
MRI	Magnetic Resonance Imaging
PAU	Penetrating Atherosclerotic Ulcer
PET	Positron Emission Tomography
TGF-β	Transforming Growth Factor Beta
US	Ultrasound
FDG	Fluorodeoxyglucose (for PET)
ROI	Region of Interest
